# Inverse regulation of Fe- and Ni-containing SOD genes by a Fur family regulator Nur through small RNA processed from 3′UTR of the *sodF* mRNA

**DOI:** 10.1093/nar/gkt1071

**Published:** 2013-11-13

**Authors:** Hae Mi Kim, Jung-Ho Shin, Yoo-Bok Cho, Jung-Hye Roe

**Affiliations:** Laboratory of Molecular Microbiology, School of Biological Sciences, Institute of Microbiology, Seoul National University, Seoul 151-742, Korea

## Abstract

Superoxide dismutases (SODs) are widely distributed enzymes that convert superoxides to hydrogen peroxide and molecular oxygen, using various metals as cofactors. Many actinobacteria contain genes for both Ni-containing (*sodN*) and Fe-containing (*sodF*) SODs. In *Streptomyces coelicolor,* expression of the *sodF* and *sodN* genes is inversely regulated by nickel-specific Nur, a Fur-family regulator. With sufficient nickel, Nur directly represses *sodF* transcription, while inducing *sodN* indirectly. Bioinformatic search revealed that a conserved 19-nt stretch upstream of *sodN* matches perfectly with the *sodF* downstream sequence. We found that the *sodF* gene produced a stable small-sized RNA species (s-SodF) of ∼90 nt that harbors the anti-*sodN* sequence complementary to *sodN* mRNA from the 5′-end up to the ribosome binding site. Absence of nearby promoters and sensitivity to 5′-phosphate-specific exonuclease indicated that the s-SodF RNA is a likely processed product of *sodF* mRNA. The s-SodF RNA caused a significant decrease in the half-life of the *sodN* mRNA. Therefore, Nur activates *sodN* expression through inhibiting the synthesis of *sodF* mRNA, from which inhibitory s-SodF RNA is generated. This reveals a novel mechanism by which antagonistic regulation of one gene is achieved by small RNA processed from the 3′UTR of another gene’s mRNA.

## INTRODUCTION

Superoxide dismutases (SODs) are ubiquitous enzymes that catalyze conversion of superoxide to molecular oxygen and hydrogen peroxide, using catalytic metal ions. Depending on their structure and metal cofactors, three classes of SODs have been reported; Cu/Zn-SOD found in eukaryotes and some bacteria, Fe- or Mn-containing SOD present in bacteria, mitochondria and chloroplasts, and Ni-containing SOD found in some bacteria (1–4). They are present both in aerobes as well anaerobes, protecting and preparing cells against superoxide toxicity in the presence of O_2_ (5). Cells usually contain more than two types of SODs in diverse combinations, whose production is regulated tightly in response to metabolic and environmental cues.

In *Escherichia coli*, where *sodA*, *sodB* and *sodC* genes encode Mn-SOD, Fe-SOD and Cu,Zn-SOD, respectively, the presence of metal, oxygen and redox-active compounds, as well as growth phase regulate their gene expression via transcriptional regulators such as Fur, Fnr, ArcA, SoxR and RpoS (6,7). Regulation of *sodA* and *sodB* genes encoding cytoplasmic SODs is intricately inter-connected by a global regulator Fur in response to iron availability. In the presence of iron, Fur represses transcription of *sodA* and small regulatory RNA RyhB that inhibits translation and stability of *sodB* RNA, allowing the production of Fe-SOD. In the absence of iron, expression of *sodA* and RyhB is induced, resulting in the production of MnSOD (8–10). This mode of regulation is conserved in Pseudomonads, where the inverse regulation of *sodA* and *sodB* genes in response to iron is exerted by Fur, and *sodB* is activated by Fur through inhibiting the transcription of small regulatory RNA PrrF1 and PrrF2, functional homologs of RyhB (11–13). In *Bacillus subtilis*, production of Fe-containing proteins is activated by Fur through repressing transcription of yet another small RNA FsrA (14). These examples support the presence of an evolutionarily robust regulatory circuit mediated by an iron-specific regulator Fur and small RNAs in coordinated synthesis of iron-requiring proteins across distantly related bacteria.

NiSOD and its encoding gene (*sodN)* were first discovered in *Streptomyces* spp. (15). The *sodN* gene was subsequently found in the genome of nearly all streptomycetes, various actinomycetes (16,17), diverse marine cyanobacteria (18–21), and some distantly related proteobacteria and eukaryotic green algae (17). The SodN protein is processed at its N-terminal region by its cognate peptidase to produce active Ni-SOD (22), which consists of homohexameric polypeptides with bound nickel at the N-terminal hook of each monomer (23,24). Bioinformatic analyses predicted the presence of NiSOD either alone in some actinobacteria and marine cyanobacteria or in combination with FeSOD, MnSOD or CuZnSOD (16,25).

In *Streptomyces coelicolor*, where Fe-SOD and Ni-SOD are present, the two enzymes are produced in an antagonistic fashion in response to the presence of nickel in the media (26,27). Expression of the *sodF* gene encoding Fe-SOD is inhibited by nickel through a nickel-specific Fur-family regulator Nur that binds to and inhibits expression from the *sodF* promoter in the presence of nickel (26,28,29). Nur also negatively regulates nickel-uptake genes, justifying its name as nickel-uptake-regulator (28). On the other hand, expression of the *sodN* gene requires Nur as a positive regulator in the presence of nickel (28). However, Nur does not bind to the *sodN* gene, most likely acting via an indirect way that needs to be revealed (28,30).

In this article, we investigated how the antagonistic regulation of *sodF* and *sodN* is achieved through Nur. We found that a small regulatory RNA is produced from *sodF* mRNA by endonucleolytic cleavage of about 90 nt from its 3′UTR. This provides a novel example of small regulatory RNA produced from the 3′UTR of functional mRNA by cleavage. Very recently, the presence of 3′UTR-generated processed small RNAs associated with Hfq in *Salmonella* has been demonstrated (31,32). However, their role as regulatory molecules, rather than intermediates in mRNA degradation pathway, awaits experimental validation. We now present the first example of a small regulatory RNA that is generated from the 3′UTR of a functional mRNA without affecting its coding region. These findings led us to propose a conclusive model where Nur activates *sodN* expression by inhibiting the production of small *sodF* RNA, which pairs with *sodN* mRNA, blocks its translation and facilitates *sodN* mRNA decay.

## MATERIALS AND METHODS

### Strains and growth conditions

*Streptomyces coelicolor* A3(2) M145 strain and its derivatives were grown and maintained according to standard procedures (33). For liquid culture, YEME medium (0.3% yeast extract, 0.3% malt extract, 0.5% peptone, 1% glucose, 10 or 34% sucrose, 5 mM MgCl_2_ separately autoclaved) was used. For nickel treatment, 50 μM NiSO_4_ was added to the culture when inoculating seed culture. The *Δnur, ΔsodF* and *ΔsodF2* strains had been previously generated in our laboratory (28,34,35).

### Construction of s-SodF overproducing strain

The *sodF* downstream fragment (from +10 to +142 nt from the end of the *sodF* stop codon) was generated from M145 chromosomal DNA by polymerase chain reaction (PCR) using primer pairs as described in Supplementary Table S1. The PCR product was fused by overlapping PCR to another 240 bp PCR-generated fragment containing the strong ermE* promoter (36). The 399-bp final PCR product was cut with EcoRI/XbaI restriction enzymes and was cloned into pSET162, an integration vector containing thiostrepton marker (37). The recombinant plasmid was confirmed by nucleotide sequencing and was then transformed into the *ΔsodF* strain. As a negative control, the parental vector pSET162 was introduced to *ΔsodF* in parallel.

### S1 mapping and northern analyses

**Figure 1.**
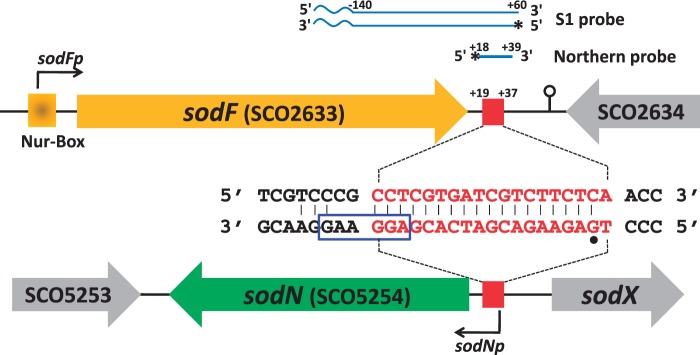
Complementarity between sense strands of *sodF* and *sodN* genes. Gene structures around *sodF* and *sodN* genes are schematically shown. The same 19 bp DNA sequence downstream and upstream of *sodF* and *sodN* genes, respectively, are shown as red square. The position of promoters (*sodFp* and *sodNp*) and Nur-binding site (Nur-box) was marked. The complementary sequence in the 3′ and 5′UTR of *sodF* and *sodN* transcripts, respectively, was shown in the middle. The position of 5′-end and ribosome binding site of *sodN* mRNA was indicated with a dot and square box, respectively. Probes for S1 mapping and northern analysis of *sodF* RNAs were presented with radiolabeled positions (asterisks) along with relative distances from the end of the stop codon of *sodF* ORF. The waved segment of S1 probe indicates non-related vector sequence.

RNAs were isolated from wild type (M145) and various mutant cells grown in YEME to OD_600_ of 0.2 to 1.5. Harvested cells were disrupted in modified Kirby mixture (33) using an ultrasonicator with a microtip at 20% of the maximum amplitude (600 W, 20 kHz). Following extraction with phenol/chloroform, the supernatant was precipitated with isopropanol. The RNA pellet was dissolved in diethyl pyrocarbonate-treated distilled water and quantified by measuring its absorbance at 260 nm. To visualize rRNAs and check for contamination by genomic DNA, RNA samples (10 μg each) were electrophoresed in 1.3% agarose gel in 3-(N-morpholino) propanesulfonic acid buffer. For S1 mapping, DNA probes for *sodN* and *sodF* transcripts were generated by PCR using M145 chromosomal DNA as a template. The PCR-generated *sodN* probe spans from −175 to +127 nt relative to the start codon of the *sodN* coding region. To generate S1 probes for *sodF* RNA, the PCR-generated *sodF* fragments (−140 to +60 nt and −205 to +141 nt relative to the end of the *sodF* stop codon) were cloned into the pGEM-T easy vector (Promega), generating pGEM-sodF200 and pGEM-sodF346, respectively. From pGEM-sodF200, the probe DNA was generated by second PCR, using a T7 forward primer and *sodF* (+60) reverse primer (Supplementary Table S1), generating a 278-bp DNA fragment containing the *sodF* gene (−140 to +60) linked to 78 bp of vector sequence (Figure 1). From pGEM-sodF346, the probe DNA was generated by PCR, using a SP6 forward primer and sodF(+78) reverse primer (Supplementary Table S1), generating a 383-bp DNA fragment containing the *sodF* gene (−205 to +78) linked to 100 bp of vector sequence. The probe DNAs were radiolabeled at their 5′-ends with [γ-^32^P]-ATP by T4 polynucleotide kinase. For each RNA sample (25 μg), probe DNA was hybridized and digested with S1 nuclease according to the standard procedures. For 3′-end mapping of the *sodF* RNA, the probe was generated by PCR from pGEM-sodF346 as a template, using T7 and SP6 primer. BssSI-cut PCR product (199 bp) containing the *sodF* gene (+21 to +141) and 78 bp of vector sequence was labeled with [α-^32^P]-dATP. The protected fragments were analyzed on a 6% polyacrylamide gel containing 7 M urea. For high-resolution S1 mapping, a sequencing ladder was generated using sequenase version 2.0 as recommended by the manufacturer (USB corporation), using the sodF(+78) oligonucleotide primer and the template pGEM-sodF346 DNA in a labeling mix with [α-^35^S]-dATP. For northern analysis of *sodF* RNAs, a 22-nt single-stranded DNA probe (+18 to +39 nt relative to the end of the *sodF* stop codon; Supplementary Table S1) was synthesized, and labeled with [γ-^32^P]-ATP by T4 polynucleotide kinase. Each sample of 70 μg total RNA was resolved on 12.5% polyacrylamide gel containing 7 M urea, and transferred to a Zetaprobe-GT-membrane (Bio-Rad). Radioactive signals were detected and quantified by phosphor screen and image analyzer (FLA-2000; Fuji).

### 5′ RACE

5′-rapid amplification of cDNA ends (RACE) was carried out using the FirstChoice® RLM-RACE kit (Ambion), following the manufacturer’s instructions with modifications for bacterial RNA (38). Briefly, 10 μg of total RNA, extracted from *S. coelicolor* M145 was treated with tobacco acid pyrophosphatase (TAP) before being ligated to a 5′-RACE adapter using T4 RNA ligase. This ligated product was then used as template for reverse transcription using primers complementary to *sodF* and *sodN*, together with SuperScript™ III reverse transcriptase (Invitrogen). The resulting cDNA then served as template for 5′-end PCR amplification, using an adaptor-specific outer primer (Supplementary Table S1) and the same oligonucleotide was used to prime the reverse transcription reaction. The second PCR was done with a pair of inner primer specific for adaptor and RNA, respectively. The final PCR products were separated on an agarose gel, excised and purified using the Qiagen® Gel extraction kit, and then sequenced.

### Exonuclease digestion of RNA

RNA samples were prepared from the wild type and *Δnur* cells grown exponentially in YEME. Treatment of RNA samples with TAP and the terminator 5′-phosphate-dependent exonuclease (5′-exo) was done as described previously (39). Total RNAs (5 μg per each sample) were incubated in 10 μl reaction volume containing 1 μl of 10X TAP reaction buffer (0.5 M sodium acetate, pH 6.0, 10 mM ethylenediaminetetraacetic acid, 1% β-mercaptoethanol, 0.1% Triton X-100), 0.5 μl of TAP (5 μ; Epicentre) or water, and 1 μl of RNasin (40 μ; Promega) for 3 h at 37°C. The reaction mixture was extracted with phenol/chloroform, and the RNA was precipitated by ethanol. Further digestion with Terminator Exonuclease (Epicenter) was carried out in 20 μl reaction volume containing 2 μl of 10X reaction buffer (500 mM Tris Cl, pH 8.0, 20 mM MgCl_2_, 1 M NaCl), 1 μl of RNasin (40 U; Promega) and 1 μl of either Terminator Exonuclease (1 U; Epicentre) or water, for 3 h at 30°C. The reaction mixture was extracted with phenol/chloroform, ethanol precipitated in the presence of glycogen and analyzed by northern blotting as described above.

## RESULTS

### Presence of complementarity between the sense strands of *sodF* and *sodN* genes

The intergenic region between the *sodN* (SCO5254) gene and the *sodX* (SCO5255) gene, where SodX encodes a cognate peptidase for SodN, is highly conserved among streptomycetes (Supplementary Figure S1). A 19-bp sequence stretch within this conserved region also appears in the downstream of the *sodF* gene (17). This 19-nt stretch is complementary between the sense strands of *sodF* and *sodN* genes (Figure 1). Previously mapped 3′- and 5′-ends of *sodF* and *sodN* mRNAs, respectively, in *S. coelicolor* Müller (26,27), led us to hypothesize that a significant fraction of the complementary sequence can actually form base pairs between the 3′UTR of *sodF* and 5′UTR of *sodN* transcripts in the *S. coelicolor* A3(2) M145 strain that we used in this study. We determined the 5′-end of the *sodN* mRNA in *S. coelicolor* by 5′ RACE, and found that it starts at the same G nucleotide as determined for the Müller strain (27) (Figure 1). The complementary base-pairing region spanned the first 18 nt of the *sodN* mRNA, encompassing part of the ribosome binding site (Figure 1). We investigated whether the complementary base pairing between *sodF* and *sodN* transcripts was responsible for the inverse regulation of the two genes, and if so, how it was mediated.

### Small-sized *sodF* RNAs encompassing the anti-*sodN* sequence are produced in a Nur-dependent manner

**Figure 2.**
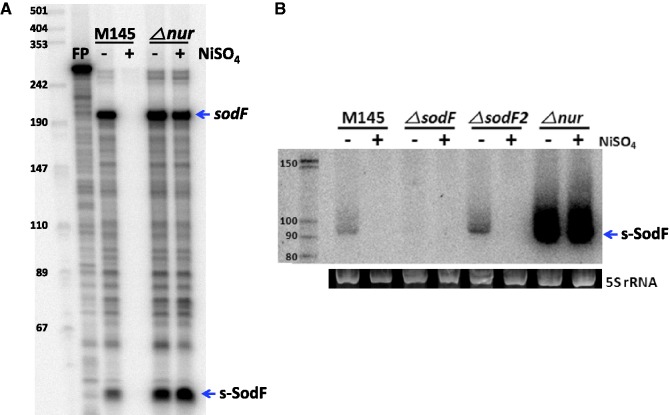
Small-sized *sodF* RNA that contains the 19 nt *sodN*-complementary (anti-*sodN*) sequence is produced in a Nur-dependent manner. (**A**) S1 mapping of *sodF* RNAs with DNA probe 5′-end labeled at +60 position relative to the stop codon of the *sodF* ORF. RNAs were prepared from the wild-type (M145) and *△nur* mutant grown in the presence and absence of 50 μM NiSO_4_. The arrows indicate the presence of *sodF* RNAs that fully protected the probe (*sodF*) and partly protected (s-SodF). FP denotes free probe with plasmid vector sequence attached. (**B**) Northern blot analysis of *sodF* RNAs. RNAs were prepared from the wild type (M145), *△sodF, △sodF2* and *△nur* cells grown in the presence and absence of 50 μM NiSO_4_. RNAs were run on 12.5% PAG to resolve small-sized RNAs and hybridized with 22 nt single-stranded DNA probe (+18 to +39 nt relative to the *sodF* stop codon) labeled at 5′-end by ^32^P (Figure 1). The 5S rRNA band electrophoresed on agarose gel was shown in parallel to demonstrate the quantity and quality of RNA samples.

Previously determined positions of the 5′- and 3′-ends of the *sodF* mRNA in *S. coelicolor* Müller strain lay 38 nt upstream from the start codon and 86 nt downstream from the stop codon, respectively (26). The 19 nt *sodN*-complementary (anti-*sodN*) sequence in M145 started 19 nt downstream from the stop codon of the *sodF* ORF (+19 to +37 relative to the end of the stop codon; Figure 1). In order to monitor the presence and the boundary of transcripts generated from the *sodF* downstream region, we performed S1 mapping and northern analyses. For S1 mapping, we used a 5′-end-labeled DNA probe that contained part of the *sodF* coding sequence and its downstream region (−140 to +60 relative to the end of the stop codon, Figure 1). This probe detected the presence of two kinds of RNA; one producing a fully protected band of ∼200 nt (*sodF*) and the other (s-SodF) protecting ∼50 nt band (Figure 2A). The amount of both RNAs decreased significantly in the wild-type (M145) cell when nickel was added to the culture and were produced in a constitutive manner in a *Δnur* mutant. The longer protecting transcript most likely corresponded to the *sodF* mRNA encoding Fe-SOD, whereas the short-protecting RNA (s-SodF) lacked the *sodF* coding sequence but could contain the anti-*sodN* sequence.

Since S1 mapping detected only the 5′-end point of the s-SodF RNA, we performed northern blot analysis to determine its size. Using a short DNA probe of 22 bp that encompassed the anti-*sodN* sequence, we detected a small RNA of about 90 nt in a 12.5% polyacrylamide gel specified for resolving small RNAs (Figure 2B). The amount of this RNA decreased in the presence of nickel and was constitutively enhanced in *Δnur* mutant, consistent with the observation by S1 mapping. The amount of s-SodF RNA was greatly reduced in a *ΔsodF* mutant. In contrast, deletion of *sodF2*, a paralog of *sodF* in *S. coelicolor* (35), did not affect the production of s-SodF, suggesting that the s-SodF RNA is primarily produced from the *sodF* gene. Northern blot analysis clearly revealed that there existed a small RNA species produced from the *sodF* downstream region, justifying the name s-SodF. The results also clearly indicated that the production of s-SodF RNA is inhibited by nickel and Nur.

### The 5′ and 3′ boundaries of s-SodF RNA

**Figure 3.**
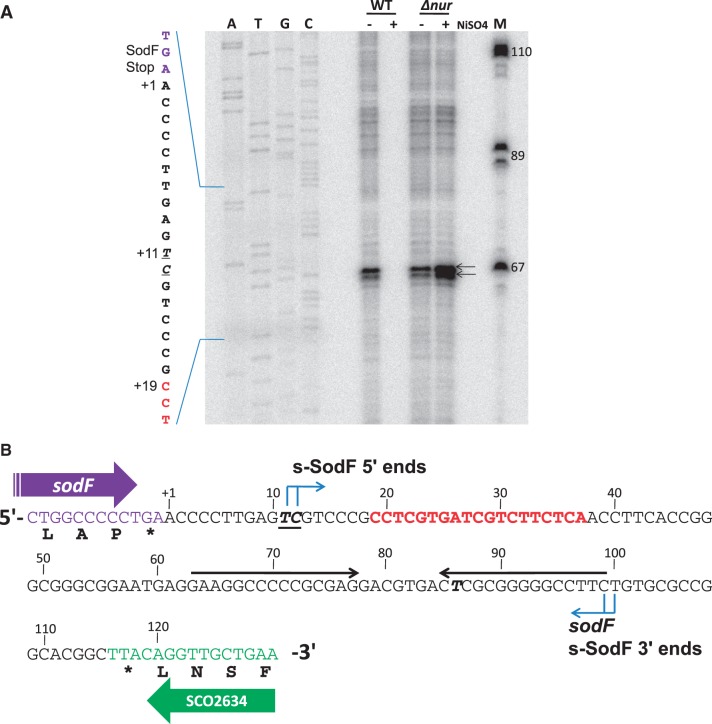

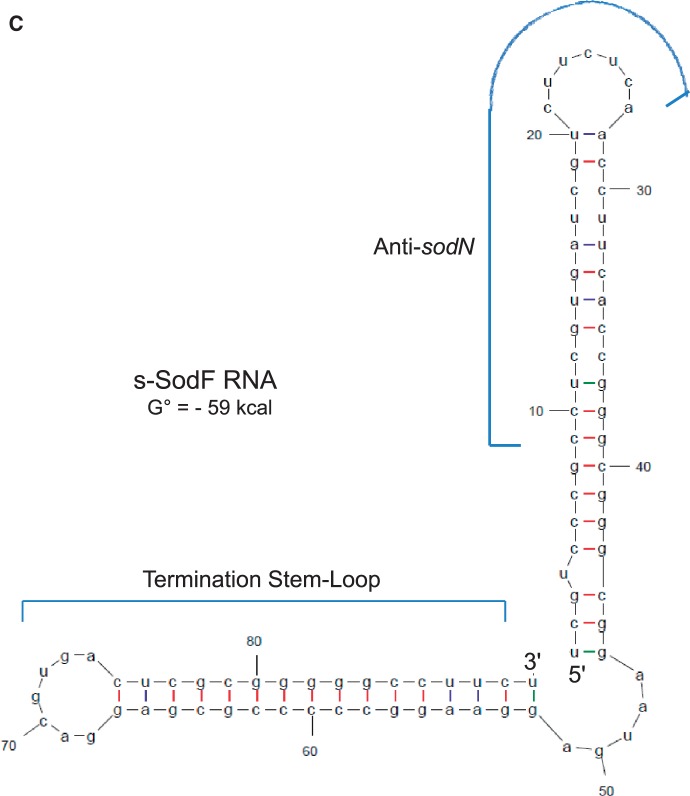
Determination of 5′-ends of s-SodF RNA. (**A**) Determination of 5′-ends by high-resolution S1 analysis. S1 mapping of *sodF* RNAs with a DNA probe 5′-end labeled at +78 nucleotide position relative to the stop codon of the *sodF* ORF. RNAs were prepared from the wild type (M145) and *△nur* mutant grown in the presence and absence of 50 μM NiSO_4_. The position of 5′-ends of the transcripts is underlined. The sequencing ladder was obtained with the *sodF* oligonucleotide primer (5′-CCT CGC GGG GGC CTT CCT CA-3′) and the template pGEM-sodF346. (**B**) Sequence information of intergenic region between *sodF* (*SCO2633*) and *SCO2634*. The nucleotide position was numbered relative to the end of the stop (TGA) codon of *sodF* ORF. The 5′-end position of s-SodF was marked (bold italic underlined) along with 19 nt anti-*sodN* sequence (red), 15 nt inverted repeats (horizontal arrows), and the possible 3′-end position of *sodF* and s-SodF RNAs. (**C**) Predicted secondary structure for s-SodF RNA. The secondary structure of 90 nt long s-SodF RNA (from +11 to +100; as in (B)), as well as stability, were predicted by mfold program (41). The 5′ proximal stem-loop contains the anti-*sodN* sequence.

In order to determine the end points and the exact size of the s-SodF RNA, we performed high-resolution S1 mapping. The 5′-end mapping was done with the probe labeled at +78. The result showed that the 5′-end of s-SodF was localized at nucleotides T and C (+11 and +12 relative to the end of the stop codon), 7–8 nt upstream of anti-*sodN* sequence (Figure 3A). The 3′-end mapping was done with the probe labeled at +21. However, we were not able to localize the exact 3′-end point because the sequencing ladder from the template DNA was compressed severely around the protected band size. This indicated the presence of stable secondary structure near the 3′-end. End-mapping with 3′ RACE was not successful either. The presence of an inverted repeat of 15 nt stretch with 80% GC suggested a stable stem and loop structure (ΔG° = −34.5 kcal), which could hinder nucleotide sequencing ladder. This stem-loop structure likely served as an instrinsic transcription terminator. This coincided with the most prevalent type of intrinsic terminators in actinobacteria, with stem and loop structure of ΔG between −15 and −25 kcal, followed by less than two U residues (40). From the approximate size estimation of s-SodF by S1 mapping and northern analysis, combined with the presence of a potential intrinsic terminator sequence, we propose that the 3′ boundary of s-SodF RNA lies at the end of the inverted repeat near +100, and thus we estimate s-SodF to be 88–90 nt long (Figure 3B).

We predicted the secondary structure of 90 nt s-SodF RNA (+11 to +100; Figure 3B) using mfold program (41). Figure 3C demonstrated that s-SodF can form a structure with two stem loops, with an overall ΔG° value of about −59 kcal. The 5′-proximal stem-loop has the anti-*sodN* sequence that spans from the middle of the stem to the entire loop. This stem-loop contains bubbles and a bulge in the stem with an estimated ΔG° of about −22 kcal. The predicted structure suggests that the 5′-end of *sodN* mRNA can easily start pairing with s-SodF through the anti-*sodN* sequence in the loop region. The termination stem-loop has a perfectly paired 16 bp stem (including the terminal G-U base pairing) with estimated ΔG° of about −36 kcal.

### The s-SodF is produced from processing of the *sodF* mRNA

The observation that s-SodF RNA is produced in a Nur-dependent manner led us to search for the presence of Nur binding sequence in the genome near the starting position of the small RNA. No sequence matching the proposed Nur-box consensus (tTGCaa-N5-ttGCAA) was found (29). Electrophoretic mobility shift assay with the 200-bp DNA probe used for S1 mapping analysis (Figure 1) did not detect any binding protein present in cell extracts prepared from wild-type cells grown in Ni-supplemented YEME medium (data not shown). Therefore, the possibility of initiating transcription from its own Nur-dependent promoter for s-SodF synthesis appeared very low.

**Figure 4.**
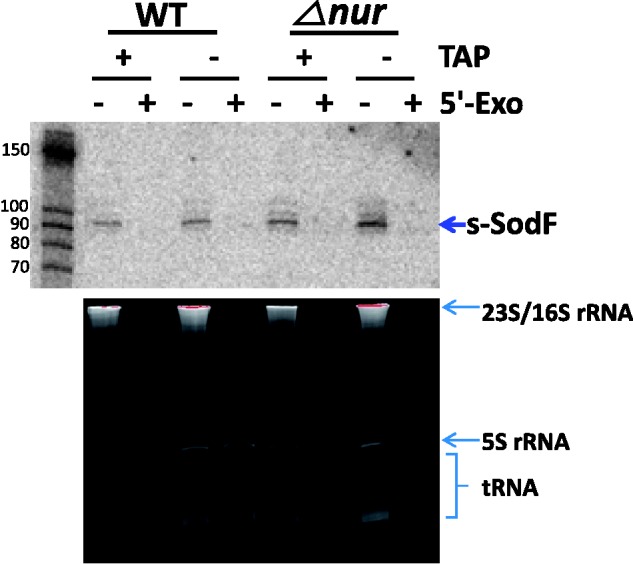
Sensitivity of s-SodF RNA toward 5′ monophosphate-dependent exonuclease. The phosphorylation state of the s-SodF RNA was monitored by treatment with a 5-monophosphate-dependent exonuclease (5′-Exo). Total RNA isolated from wild type (M145) and *△nur* cells were subjected to digestion with 5′-Exo and analyzed by gel electrophoresis (lower panel) and northern blotting with an s-SodF specific radiolabeled probe (upper panel). RNA samples were treated either with or without TAP that converts 5′-triphosphorylated RNA to 5′-monophosphorylated one prior to 5′-Exo treatment. The status of stable RNAs in each sample is shown in the lower panel.

The 5′ phosphorylation status of s-SodF RNA was then examined using 5′-phosphate-dependent exonuclease. Total RNAs isolated from either wild type (M145) or *Δnur* mutant cells were treated with 5′-monophosphate-dependent exonuclease as described previously (39), and subjected to 12.5% PAGE and northern analysis. Results in Figure 4 (upper panel) demonstrated that 5′ exonuclease digested s-SodF RNA efficiently as it did the bulk of 23 S and 16 S rRNAs (bottom panel). Treatment with TAP that removes pyrophosphates from 5′ triphosphates of *de novo* transcribed bacterial mRNA did not make any difference. Therefore, it is highly likely that the 5′-end of s-SodF RNA is monophosphorylated, and hence was generated not by *de novo* transcription but from the cleavage of a longer RNA. Since the *sodF* mRNA spanned not only the coding region but also the downstream UTR that encompasses the anti-*sodN* sequence, it was most likely that s-SodF is generated from the full-length *sodF* mRNA by cleavage at the 5′ side of T (+11) or C (+12) residues (Figure 3).

### s-SodF RNA decreases the stability of *sodN* mRNA

**Figure 5.**
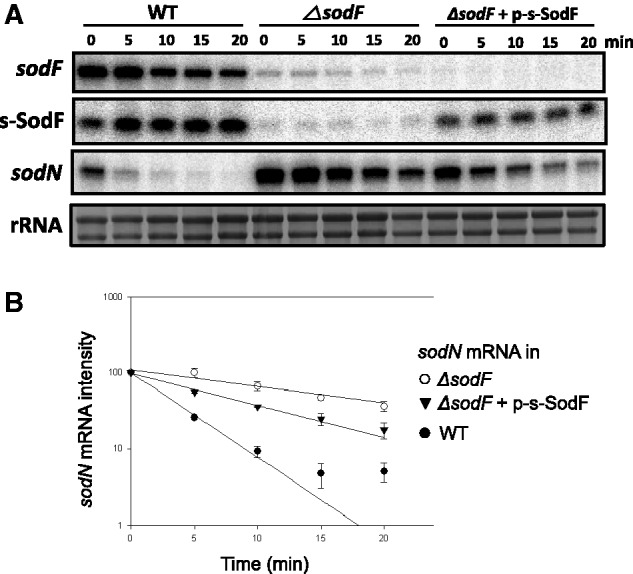
Effect of s-SodF RNA on the stability of *sodN* mRNA. (**A**) S1 mapping of *sodF* and *sodN* RNAs following rifampicin treatment. Wild-type (M145) and *△sodF* cells with or without pSET-based recombinant plasmid expressing s-SodF RNA from ermE* promoter (p-s-SodF) in the chromosome were grown in YEME medium to OD600 of 0.8 and treated with rifampicin (300 μg/ml). At 5, 10, 15 and 20 min after rifampicin treatment, cells were harvested and fixed with methanol. RNA samples were analyzed by S1 mapping with 5-end-labeled probes. A representative result was presented. (**B**) The relative amount of each RNA species was plotted to estimate half-life.

We then explored whether s-SodF RNA affected *sodN* gene expression. We estimated the half-life of *sodN* mRNA under various genetic background. Depending on growth conditions, the ratio of *sodF* to *sodN* mRNAs varied (see below). We measured the RNA decay rates, following the inhibition of transcription initiation by rifampicin. Cells grown in YEME to OD_600_ of 0.8 were treated with rifampicin (300 μg/ml), and were harvested at different time points (0–20 min) to prepare RNA. S1 mapping of *sodF* mRNA, s-SodF and *sodN* mRNA in each sample was done with RNA-specific probes in one reaction tube. As demonstrated in Figure 5, the *sodN* mRNA in the wild-type strain decayed at t_1/2_ of ∼3 min. The *sodF* mRNA decayed with half-life of about 10 min, whereas the s-SodF RNA was relatively stable with t_1/2_ of >>20 min. In *ΔsodF* mutant where no *sodF* transcripts are produced, the half-life of *sodN* mRNA increased to about 16 min, suggesting that *sodF* transcripts decrease the stability of *sodN* mRNA. A residual *sodF*-sized band observed in *ΔsodF* mutant is thought to be non-specific, and it is not detectable in the mutant introduced with pSET-derived plasmids (see below).

In order to verify the effect of s-SodF RNA alone, we introduced to *ΔsodF* mutant an overexpression plasmid for s-SodF RNA whose expression was driven by a strong ermE* promoter on the integrating vector pSET162. Figure 5 demonstrated that when s-SodF RNA was stably expressed in the absence of *sodF* mRNA, the half-life of *sodN* mRNA was about ∼7 min which was significantly shorter than ∼16 min observed in *ΔsodF*. Since the expression level of s-SodF RNA in *ΔsodF* was lower than the level in the wild type, it is thought that the *sodN* half-life was not brought down to the wild-type level (∼3 min). This experiment clearly demonstrated that the production of s-SodF RNA alone was sufficient to decrease the amount of *sodN* mRNA.

### Mutations in the anti-*sodN* region of s-SodF inactivate its inhibitory function

**Figure 6.**
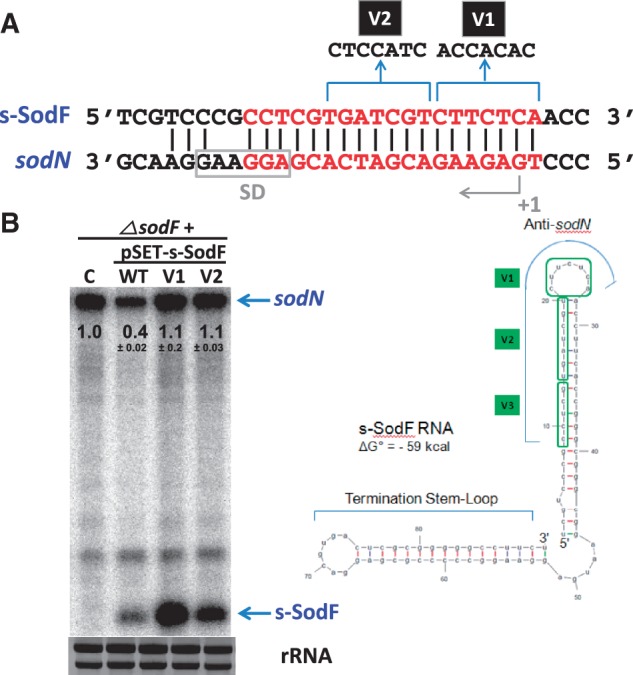
Sequence-specificity of s-SodF RNA to inhibit *sodN* expression. (**A**) Variants of s-SodF were created by changing sequences in the anti-*sodN* region. V1 harbor changes of seven nucleotides that correspond to the predicted loop region in anti-*sodN* sequence. V2 harbor changes in the subsequent seven nucleotides. (**B**) Effect of s-SodF mutations on the level of *sodN* mRNA. The mutated s-SodF genes were cloned in pSET152-based vector pSET162 with ermE* promoter, and incorporated into the chromosome of *ΔsodF* strain through phage attachment site, as done for the wild-type s-SodF construct used in Figure 5. The *ΔsodF* cells with parental vector control (**C**), wild type (WT) and variants (V1, V2) of s-SodF genes were grown in YEME to OD of 0.8. RNA samples were obtained from cells as in Figure 5, without rifampicin chase. A representative gel from three independent experiments was presented, and marked with the quantified values of the average ± SD for *sodN* mRNA.

In order to test whether the anti*-sodN* sequence in s-SodF was indeed critical to reduce the amount of *sodN* mRNA, we created mutants in this region. Variants with changes in the predicted 7-nucleotide loop region (V1), and the subsequent seven nucleotides (V2) were made by site-directed mutagenesis (Figure 6) using mutagenic primers. The secondary structure prediction by mFOLD suggested that V1 would assume almost identical structure to the wild type with ΔG° of −59 kcal, and V2 a similar structure with a larger loop with ΔG° of −55 kcal. We cloned the mutant s-SodF genes in the integration vector (pSET162) and introduced them into the chromosome of *ΔsodF* strain of *S. coelicolor,* in the same way as we made the expression construct for the wild type s-SodF (Figure 5). The results demonstrated that the introduction of wild-type s-SodF decreased the level of *sodN* mRNA to about 40% of that in the control strain without any s-SodF gene. On the other hand, V1 and V2 variants of s-SodF, even though they were expressed to higher levels than the wild type, did not affect the level of *sodN* mRNA (Figure 6B). The levels of V1 and V2 s-SodF RNAs were about 7-fold and 3-fold higher than the wild-type level, respectively. This differential expression could arise from the difference in the copy number of incorporated plasmids as well as RNA stability. We tried another stem-variant mutant (V3) that changed the last five nucleotides in the anti-*sodN* sequence from CCTCG to AACAT. This variant, however, was expressed too low to examine its effect, presumably due to a dramatic decrease in stability, as expected from the central bulge generated in the stem (data not shown). All together, these experiments verify that sequences in the *anti-sodN* region of s-SodF, at least the first 14 nt including the predicted loop, were critical for the inhibitory action of s-SodF.

### Growth phase-dependent antagonistic expression of *sodN* and *sodF*

**Figure 7.**
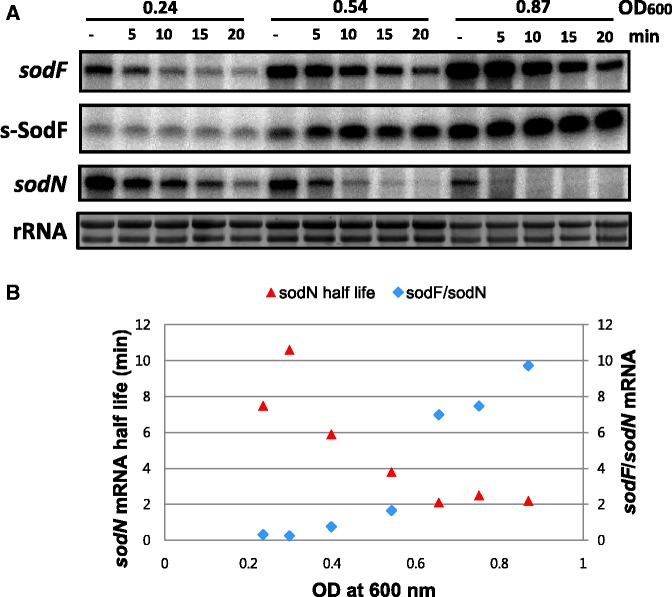
Correlation between *sodF/sodN* ratio and the stability of *sodN* mRNA during growth. (**A**) Measurement of *sodF* and *sodN* mRNA stability at different growth phases in WT. *S. coelicolor* cells were grown in YEME medium to OD_600_ of 0.2 to 1.0 and treated with rifampicin (300 μg/ml) at specific OD_600_. At 5, 10, 15 and 20 min after rifampicin treatment, cells were harvested and fixed with methanol. Their RNAs were analyzed by S1 mapping with 5′-end-labeled probes for *sodF* and *sodN* RNAs. The radioactivity of the S1-protected bands was quantified by Phosphor Imager (Bio-Rad) and presented as relative values to the untreated sample. Representative results from cells grown to OD_600_ of 0.24, 0.54 and 0.87 were shown. (**B**) The change in the relative amount of *sodF* and *sodN* mRNA (*sodF/sodN*) during exponential growth was plotted, along with the changing half-lives of *sodN* mRNA, as batch culture proceeds. The *sodF/sodN* ratio was taken from the quantified amount of each mRNA-specific band in untreated samples at specific growth stage, as represented in (A).

When we tried to measure the half-life of *sodN* mRNA in the wild-type strain, we experienced extensive variation. Soon we found that the amount of *sodN* mRNA decreased, whereas *sodF* RNA increased as cell growth proceeded from early to late stages of exponential growth. At early exponential phase (OD_600_ < 0.5), *sodN* RNA was more prevalent than *sodF* RNA, whereas the relative amount reversed at later growth phase (Supplementary Figure S2). We then measured the half-life of *sodN* mRNA at different OD by rifampicin chase experiment. A representative result presented in Figure 7 demonstrated clearly that at later phase of growth, where the amount of *sodF* RNA increased, the half-life of *sodN* mRNA became shorter. We plotted the change in *sodN* mRNA half-life as growth progresses in the exponential phase, along with the relative amount of *sodF* mRNA to *sodN* mRNA. Results in Figure 7B coincided well with the proposal that *sodF* transcripts lower the stability of *sodN* mRNA.

## DISCUSSION

### Small regulatory RNA produced from a functional mRNA inhibits the expression of an antagonistically regulated gene

**Figure 8.**
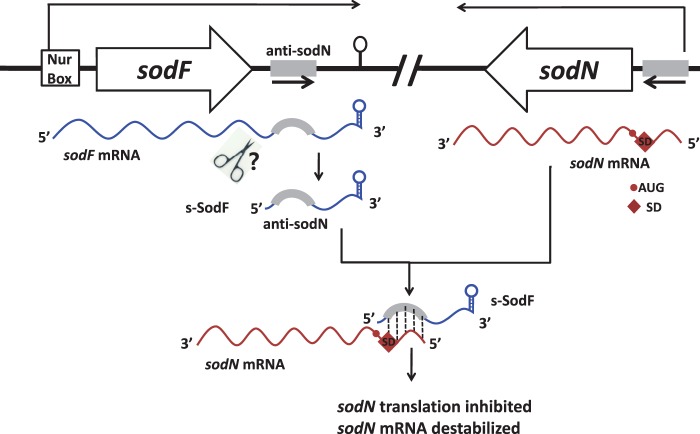
A model for Nur-dependent inverse regulation of *sodF* and *sodN.* This model describes that the small processed *sodF* RNA negatively regulates the translation and stability of *sodN* mRNA, enabling the antagonistic regulation of *sodN* and *sodF* genes through nickel-specific Nur. Under nickel-limited conditions, Nur without nickel loses its binding activity to *sodF* promoter. Induction of *sodF* gene transcription ensues, producing full length *sodF* mRNA, from which a 90 nt-long 3′UTR segment that contains anti-*sodN* sequence of 19 nt can be cleaved off to function as a stable small regulatory RNA (s-SodF). The s-SodF RNA is able to form perfect base pairing with the 5′-end segment of *sodN* mRNA by up to 18 bp. The base-pairing can inhibit translation by occluding ribosome binding, and facilitates degradation of *sodN* mRNA, resulting in rapid decrease in the production of Ni-SOD.

Accumulating lists of small non-coding RNAs have been shown to play a variety of regulatory roles (42,43). In many cases, especially in Gram-negative bacteria, these sRNAs function in association with the RNA-binding modulator Hfq to control expression of single to multiple genes, showing a broad range of specificity. Actinomycetes, Gram-positive bacteria with high GC content, along with cyanobacteria and deinococci, do not contain any apparent Hfq-homologues (38,44). Quite a number of small RNAs have been identified in actinomycetes, primarily in streptomycetes and mycobacteria, through bioinformatics combined with experimental verifications, cloning of isolated small RNAs and genome-scale deep sequencing of RNA (38,45–48). Even though some correlation with growth phases and differentiation have been demonstrated, direct targets and physiological functions of small RNAs from actinomycetes have been identified only in a very limited number of examples (46,49). This reflects difficulties not only in identifying functional small regulatory RNAs but also in predicting and/or validating their physiological targets in bacteria with genomes of high GC content. Based on sequence complementarity, we were able to identify a unique trans-acting small RNA, a cleaved 3′UTR product of a functional mRNA, which facilitates the decay of another mRNA and possibly inhibits translation, resulting in antagonistic regulation. The model depicted in Figure 8 summarized the antagonistic regulatory mechanism of *sodF* and *sodN* gene expression. According to this model, under nickel-limited conditions as experienced in later phases of growth in batch culture, Nur without corepressor nickel loses its binding affinity for the Nur-consensus (Nur-box) sequence that overlaps the *sodF* promoter (28). Induction of *sodF* gene transcription ensues, producing full-length *sodF* mRNA, from which a 90 nt-long 3′UTR segment that contains the anti-*sodN* sequence of 19 nt can be cleaved off to function as a stable small regulatory RNA (s-SodF). The s-SodF RNA is able to form perfect base pairing with the 5′ terminal portion of *sodN* mRNA by up to 18 bp. The base-pairing facilitates degradation of *sodN* mRNA and may possibly inhibit translation by occluding ribosome binding, resulting in a rapid decrease in the production of Ni-SOD. In this way, cells can ensure production of the non-nickel enzyme (Fe-SOD) and at the same time rapidly turn off the synthesis of nickel-requiring enzyme (Ni-SOD).

### Features of s-SodF RNA and possible mechanism of regulation

Most small non-coding RNAs identified so far are transcribed as independent units from their own promoter to the terminator (42,50). In some cases, the transcribed small RNA undergoes further processing to result in a more stable and/or functional form (51,52). Recently, deep sequencing of Hfq-bound small RNAs in *Salmonella* revealed that a significant proportion of them are produced from the 3′UTR (downstream of the coding region) of a transcribed gene (31). This raised the possibility of finding sRNAs which are transcribed in parallel with the mRNA downstream of the coding region, or are processed from existing mRNA. In one characterized example in *Salmonella*, DapZ was found to be transcribed from its own promoter located downstream of the coding region of *dapB*, which encodes a lysine biosynthetic enzyme (31). DapZ small RNA expression is independent of *dapB* expression, and it acts to translate at least two mRNAs encoding ABC transporters. A few sRNAs were suggested to be processed from existing mRNAs, based on the lack of promoters and the presence of 5′ monophosphate ends. However, all the cleavages sites occurred in the coding region, making the parental mRNAs inactive (31). Currently, s-SodF RNA is the only example that we know of to be produced as a processed 3′UTR product without affecting its corresponding coding sequence.

The predicted secondary structure of s-SodF RNA (Figure 3C) revealed two stem-loops, the 5′-proximal one with anti-*sodN* sequence and the termination stem-loop. The *anti-sodN* sequence was located in such a way that the final 6 nt that could pair with the 5′ terminal residues of *sodN* mRNA is present in the loop region. Therefore, it was plausible to speculate that the seed-pairing between s-SodF and *sodN* mRNA occurs through this single-stranded loop region. The stem region where the rest of the complementary sequence resides consists of relatively weak base-pairing with bubbles, easily breakable to form hydrogen bonds with *sodN* mRNA. Loss of inhibitory activity by changing sequences in the predicted loop region (V1) and the subsequent stem region (V2) predicts that base-pairing of at least 14 bp may be required for the inhibitory function of s-SodF (Figure 6). Whether the base pairing between the two molecules requires any RNA-binding protein modulators or not awaits further investigation. In *S. coelicolor*, an example has been reported for the interaction of a trans-acting small RNA (scr5239) and its target (*dagA* mRNA encoding an agarase), where a base-pairing was suggested to involve a 17 nt-long near-perfect match with a bulge of 1 nt (49). For RNAs with GC content of >75%, strict base pairing between sRNA and mRNA may be required to compete with prevalent intramolecular secondary structures with low ΔG values. The search for relatively long complementary sequence stretches in the genome may increase the possibility of identifying possible interacting partners of sRNAs. The RNases that are responsible for processing s-SodF RNA and degrading *sodN* mRNA await further investigation. Our preliminary experiments showed that mutation of genes for RNase III (53) and RNase E (54) did not affect the production of s-SodF and stability of *sodN* mRNA, suggesting the involvement of other RNases (data not shown).

### Predicted occurrence of similar regulation

From public genome databases, it is predicted that several groups of bacteria possess both *sodN* and *sodF* genes. They include most *Streptomyces* spp., some actinomycetes, cyanobacteria, chlamydiae, planctomycetes, gamma-, delta- and alpha-proteobacteria (Supplementary Table S2). Except *S. cattleya* that contains only the *sodN* gene, all 10 *Streptomyces* species whose genomes were examined exhibited the complementary sequence between the sense strands of *sodF* and *sodN* genes. They range from 16 to 19 nt of complementarity between *sodF* 3′UTR and *sodN* 5′UTR (Supplementary Table S3). Whether these other streptomycetes produce a similar s-SodF RNA and regulate *sodN* in a similar fashion as in *S. coelicolor* is an interesting question to be investigated. A recent genome-wide analysis of non-coding RNAs in three *Streptomyces* spp. revealed the presence of s-SodF RNA in *S. coelicolor* and its homologs in *S. avermitilis* and *S. venezuelae* (48). This supports the possibility that similar regulation will be in action in these organisms. In *S. bingchenggensis* BCW-1, there exist two *sodF* paralog genes as in *S. coelicolor* (Supplementary Table S3). However, unlike the two *sodF* genes (*sodF* and *sodF2*) which encode functional SodF proteins in *S. coelicolor*, only one gene (*sodF*; SBI_02504) in *S. bingchenggensis* encodes a functional SodF, followed by a degenerate anti-*sodN* sequence with 8 nt mismatch, which is unlikely to serve an inhibitory function. On the other hand, the *sodF2* gene (SBI_01257) has a big in-frame deletion of the coding region but maintains a relatively good anti-*sodN* sequence (Supplementary Table S3). If there are no sequencing errors, this suggests that the regulatory pathway with s-SodF may have been maintained throughout the evolution of *Streptomyces* spp. even when the adjacent coding sequence no longer produces a functional SodF protein. Whether there are other gene pairs or regulons that utilize this type of 3′UTR-generated small regulatory RNA requires further bioinformatic and experimental investigations. Search for complementarity between distant regions in the genome, especially between 3′UTR regions and anywhere around distant coding region, is expected to give fruitful clues. This will facilitate excavating small regulatory RNAs with putative targets.

### Inverse regulation of isoenzymes and antagonistic proteins

Antagonistic regulation between Fe-containing and Ni-containing isoforms of enzymes is not unprecedented. In *Helicobacter mustelae*, a carnivore-colonizing species, Fe-containing urease (UreA2B2) is produced under nickel-depleted condition, whereas Ni-containing urease (UreAB) prevails under nickel-sufficient condition (55). In this case, the nickel-specific regulator NikR regulates both operons, directly repressing and activating *ureA2B2* and *ureAB* transcription, respectively (56). It is interesting to note the existence of inverse regulation between FeSOD and its iso-enzymes with different metals, MnSOD and NiSOD. Since SODs are abundant proteins and are important for air-exposed life, antagonistic regulation in response to the availability of specific preferred metal and oxidative conditions should be beneficial for the economy of the cell. For soil-dwelling streptomycetes, abundant nickel in the aerobic soil environment would fit the evolution of a gene regulatory system where *sodF* is repressed such that nickel is utilized before iron. For facultative *E. coli*, life under anaerobic iron-rich condition could have evolved a gene system where SodB is a preferred enzyme under anaerobic condition, and SodA is induced in the presence of oxygen and oxidative stress.

The observation that the inverse regulation exerted by Fur family regulators involves small regulatory RNAs as mediators is intriguing. RyhB-mediated regulation by Fur in *E. coli*, PrrF1/F2-mediated regulation by Fur in *Pseudomonas* and FsrA-mediated regulation by Fur in *B. subtilis* all enable inverse regulation of Fe- versus non-Fe proteins. The discovery of s-SodF in *S. coelicolor* adds to this list of small RNA-coupled regulation by Fur family members. These examples support the presence of an evolutionarily robust regulatory circuit mediated by metal-specific Fur family regulators and small RNAs in coordinated synthesis of iso-proteins with specific metal cofactors across distantly related bacteria.

## SUPPLEMENTARY DATA

Supplementary Data are available at NAR Online, including [57–58].
